# Regorafenib inhibits tumor progression through suppression of ERK/NF-κB activation in hepatocellular carcinoma bearing mice

**DOI:** 10.1042/BSR20171264

**Published:** 2018-05-08

**Authors:** Mao-Chi Weng, Mei-Hui Wang, Jai-Jen Tsai, Yu-Cheng Kuo, Yu-Chang Liu, Fei-Ting Hsu, Hsin-Ell Wang

**Affiliations:** 1 Department of Biomedical Imaging and Radiological Sciences, National Yang-Ming University, Taiwan; 2 Division of Isotope Application, Institute of Nuclear Energy Research, Atomic Energy Council, Taiwan; 3 Division of Gastroenterology, Department of Medicine, National Yang-Ming University Hospital, Yilan, Taiwan; 4 Radiation Oncology, Show Chwan Memorial Hospital, Changhua, Taiwan; 5 Department of Radiological Technology, China Medical University, Taichung, Taiwan; 6 Department of Radiation Oncology, National Yang-Ming University Hospital, Yilan, Taiwan; 7 Department of Radiology, School of Medicine, College of Medicine, Taipei Medical University, Taipei, Taiwan; 8 Department of Medical Imaging, Taipei Medical University Hospital, Taipei, Taiwan; 9 Research Center of Translational Imaging, College of Medicine, Taipei Medical University, Taipei, Taiwan; 10 Graduate Institute of Cancer Biology and Drug Discovery, College of Medical Science and Technology, Taipei Medical University, Taipei, Taiwan

**Keywords:** apoptosis, Bioluminescence imaging, hepatocellular carcinoma, nuclear factor kappaB, Regorafenib

## Abstract

Regorafenib has been demonstrated in our previous study to trigger apoptosis through suppression of extracellular signal-regulated kinase (ERK)/nuclear factor-κB (NF-κB) activation in hepatocellular carcinoma (HCC) SK-Hep1 cells *in vitro*. However, the effect of regorafenib on NF-κB-modulated tumor progression in HCC *in vivo* is ambiguous. The aim of the present study is to investigate the effect of regorafenib on NF-κB-modulated tumor progression in HCC bearing mouse model. pGL4.50 luciferase reporter vector transfected SK-Hep1 (SK-Hep1/*luc2*) and Hep3B 2.1-7 tumor bearing mice were established and used for the present study. Mice were treated with vehicle or regorafenib (20 mg/kg/day by gavage) for 14 days. Effects of regorafenib on tumor growth and protein expression together with toxicity of regorafenib were evaluated with digital caliper and bioluminescence imaging (BLI), *ex vivo* Western blotting immunohistochemistry (IHC) staining, and measurement of body weight and pathological examination of liver tissue, respectively, in SK-Hep1/*luc2* and Hep3B 2.1-7 tumor bearing mice. The results indicated regorafenib significantly reduced tumor growth and expression of phosphorylated ERK, NF-κB p65 (Ser536), phosphorylated AKT, and tumor progression-associated proteins. In addition, we found regorafenib induced both extrinsic and intrinsic apoptotic pathways. Body weight and liver morphology were not affected by regorafenib treatment. Our findings present the mechanism of tumor progression inhibition by regorafenib is linked to suppression of ERK/NF-κB signaling in SK-Hep1/*luc2* and Hep3B 2.1-7 tumor bearing mice.

## Introduction

Human hepatocellular carcinoma (HCC), or primary malignancy of the liver, is notorious for the high mortality rate [1]. Progression of HCC is characterized by several cancer hallmarks such as sustained cell proliferation, evading apoptosis, and induction of angiogenesis and metastasis [2]. Many proliferative, antiapoptotic, angiogenic, and metastatic proteins are overexpressed and associated with poor prognosis in HCC patients [3,4].

Nuclear factor-κB (NF-κB), a transcription factor, modulates immunity, inflammation, and tumorigenesis through expression of NF-κB target genes. Activation of NF-κB can be triggered by upstream kinases and lead to expression of proliferative, antiapoptotic, angiogenic, and metastatic proteins in cancer cells [5]. Constitutive activation of NF-κB signal pathway was observed in HCC and may be a therapeutic target for HCC [6]. Many anti-HCC agents not only induce cytotoxicity but also activate NF-κB signal pathway resulting in limitation of therapeutic efficacy [7]. Inhibition of NF-κB signal pathway can block tumor progression in HCC *in vitro* and *in vivo* [8]. Therefore, patients with HCC may obtain benefit from development of inhibitors of NF-κB signal pathway.

Regorafenib (Stivarga), an analog of sorafenib, has been approved to treat HCC to prolong survival in patients who progressed on sorafenib treatment [9]. Previous studies indicate regorafenib induces apoptosis and inhibits metastatic potential through suppression of NF-κB activation in HCC cells *in vitro* [4,7]. However, whether regorafenib reduces NF-κB-modulated tumor progression in HCC *in vivo* needs to be elucidated. The aim of the present study is to investigate the effects of regorafenib on NF-κB-modulated tumor progression in SK-Hep1 hepatocellular carcinoma bearing mice. Effects of regorafenib on tumor growth and expression of NF-κB modulated angiogenic, metastatic, proliferative, and antiapoptotic proteins were evaluated by using caliper, bioluminescence imaging (BLI), *ex vivo* Western blotting, and immunohistochemistry (IHC) staining. The toxicity of regorafenib was determined with body weight of mice and hematoxylin and eosin (H&E) staining of liver sections.

## Materials and methods

### Reagents and antibodies

Regorafenib was obtained from Bayer Health Care Pharmaceuticals (Whippany, NJ, U.S.A.). Dulbecco’s modified Eagle’s medium (DMEM), fetal bovine serum (FBS), L-glutamine, and penicillin–streptomycin (PS) were bought from Gibco/Life Technologies (Carlsbad, CA, U.S.A.). Hygromycin was bought from Santa Cruz Biotechnology (Santa Cruz, CA, U.S.A.). JetPEI™ transfection reagent was purchased from Polyplus Transfection (Sélestat, Bas-Rhin, France). D-luciferin was bought from Promega (Madison, WI, U.S.A.). IHC Select HRP/DAB kit was purchased from Merck Millipore (Darmstadt, Hessen, Germany). Primary antibodies of NF-κB p65 (Ser536), P-AKT (Ser473), T-AKT, cellular FADD-like IL-1β-converting enzyme (FLICE)-inhibitory protein (C-FLIP), Cyclin-D1, Caspase-3 and Caspase-9 were bought from Cell Signaling Technology (Beverly, MA, U.S.A.). Primary antibodies of X-linked inhibitor of apoptosis protein (XIAP), TATA-binding protein (TBP), and Caspase-8 were purchased from Thermo Fisher Scientific (Fremont, CA, U.S.A.). Primary antibodies for matrix metallopeptidase (MMP-9) and vascular endothelial growth factor (VEGF) were bought from EMD Millipore (Billerica, MA, U.S.A.). Primary antibodies of phosphorylated extracellular signal-regulated kinase (P-ERK), T-ERK, induced myeloid leukemia cell differentiation protein (MCL-1), and Caspase-9 were purchased from Merck Millipore (Billerica, MA, U.S.A.), BioVision (Milpitas, CA, U.S.A.), and Proteintech (Chicago, IL, U.S.A.) respectively.

### Cell culture

HCC SK-Hep1 cells were obtained form by professor Jing-Gung Chung at Department of Biological Science and Technology, China Medical University, (Taichung, Taiwan) and used for the present study. HCC Hep3B 2.1-7 cells were purchased from Bioresource Collection and Research Center, Food Industry Research and Development Institute, Taiwan. Cells were both maintained in DMEM containing 10% FBS, PS (100 U/ml and 100 μg/ml), and 2 mM L-glutamine and incubated at 37°C in a 95% air and 5% CO_2_ humidified atmosphere [10]. pGL4.50 luciferase reporter vector transfected SK-Hep1 (SK-Hep1/*luc2*) were cultured in the same condition with addition of 100 μg/ml hygromycin.

### Plasmid transfection and stable clone selection

pGL4.50 luciferase reporter (pGL4.50[luc2/CMV]) vector was purchased from Promega (Madison, WI, U.S.A.). SK-Hep1 cell was transfected with pGL4.50 [luc2/CMV] vector using JetPEI™ transfection reagent as described previously [7]. SK-Hep1/*luc2* cells stably expressing luciferase were established by selection with adding 200 μg/ml hygromycin for 2 weeks.

### Animal study

**Figure 1:**
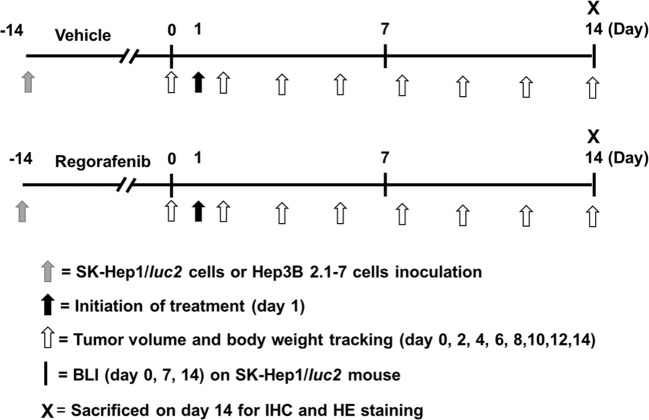
Schematic depiction of experimental protocol Please refers to “Material and methods” section in context for detail; HE, hematoxylin and eosin; IHC, immunohistochemistry.

Animal study was approved by The Institutional Animal Care and Use Committee (IACUC) in Taipei Medical University, Taipei, Taiwan (IAUCU number: LAC-2016-0029). Four-week-old nude mice were obtained from the National Laboratory Animal Center, Taipei, Taiwan. SK-Hep1/*luc2* (1 × 10^7^) or Hep3B 2.1-7 cells (2 × 10^7^) in 150 μl of mixture containing serum-free DMEM and matrigel (2:1) were inoculated subcutaneously into the right legs of nude mice [11]. When tumor volume reached about 200 mm^3^, mice were randomized into two groups (*n* = 5 for each group), vehicle group [treated with 140 μl of phosphate-buffered solution (PBS) plus 10 μl of dimethyl sulfoxide (DMSO) by gavage daily for 14 days] and regorafenib group (treated with 20 mg/kg/day by gavage for 14 days) (Figure 1). Treatment was initiated on day 1. Tumor volume was measured by digital caliper and calculated using formula 0.523 × length × width × thickness. Tumor growth was also monitored with BLI. The body weights of mice were measured after treatment together with tumor volume measurement. Mice were killed on day 14 for IHC staining of tumor tissues and pathologic examination of the liver. All animal experiments were repeated three times and complied with institutional animal care guidelines.

### *In vivo* bioluminescent imaging (BLI)

Mice were anesthetized using 1–3% isoflurane and injected intraperitoneally with 150 mg/kg D-luciferin 15 min before acquiring image. The photons emitted from tumor were detected with Xtreme (Bruker, Billerica, MA, U.S.A.). The image acquisition period was 10 s. Regions of interest (ROIs) of tumor were drawn and quantified by using Molecular Imaging Software (MI) version 7.2 (Bruker, Billerica, MA, U.S.A.) [8].

### Immunohistochemistry (IHC) staining

Mice were killed on day 14 after treatments. Tumors were removed from mice and fixed in 4% paraformaldehyde (PFA) at 4°C. Paraffin-embedded tumor tissues were sectioned in 5 μm-thick slices by Bio-Check Laboratories Ltd (New Taipei City, Taiwan). Protein levels in tumor tissues were evaluated with IHC staining. The instructions provided with the kit were followed to perform IHC staining. The sectioned slices were immunohistochemically stained with MMP-9, VEGF, MCL-1, XIAP, C-FLIP, Cyclin-D1, anti-P-ERK, NF-κB p65 (Ser536), P-AKT (Ser473), Caspase-9, -8, and -3 antibodies respectively. The stained slides were scanned using the microscopy-based TissueFAXS platform (TissueGnostics, Vienna, Austria) and images were captured at 100× magnification [12]. ImageJ software version 1.50 (National Institutes of Health, Bethesda, MD, U.S.A.) was used to evaluate indices of positivity on IHC slides.

### *Ex vivo* Western blotting

Proteins were extracted from mice tumors using nuclear and cytoplasmic extraction kits (Chemicon; EMD Millipore). Equal amount proteins were loaded on 10% sodium dodecyl sulfate/PAGE for electrophoresis assay under 100 voltage. After running step, proteins were transferred onto 0.2 μm polyvinylidene difluoride membrane and then blocking with 3% bovine serum albumin for 1 h. Primary antibodies of P-ERK (Thr202/Tyr204), T-ERK, NF-κB p65 (Ser536), P-AKT (Ser473), T-AKT, caspase-3, caspase-8 and caspase-9 were added on membrane and both were gently shaked on shaker in 4°C refrigerator overnight. The levels of protein were finally colored by enhanced chemiluminescence (ECL) and detected by ChemiDoc MP Imaging System (Bio-Rad Laboratories, Inc., CA, U.S.A.). Quantification of protein level was performed by Bio-Rad Image Lab [13].

### Pathological examination

Mice were sacrificed on day 14 after treatments. Lobes of each mouse liver were removed and fixed in 4% PFA at 4°C. Paraffin-embedded liver tissues were sectioned in 5 μm-thick slices and then stained with hematoxylin and eosin (HE) by Bio-Check Laboratories Ltd (New Taipei City, Taiwan). The stained slides were scanned using the microscopy-based Tissue FAXS platform (TissueGnostics, Vienna, Austria) and images were captured at 100× magnification.

### Statistical analysis

Student’s *t*-test was used to verify significance of difference between treatment and control group. Data presented were means ± standard error. Statistical significance was reached when *P*-value was less than 0.05. All experiments were repeated independently for three times.

## Results

### Regorafenib reduces tumor growth in SK-Hep1/luc2 and Hep3B 2.1-7 tumor bearing mice

**Figure 2:**
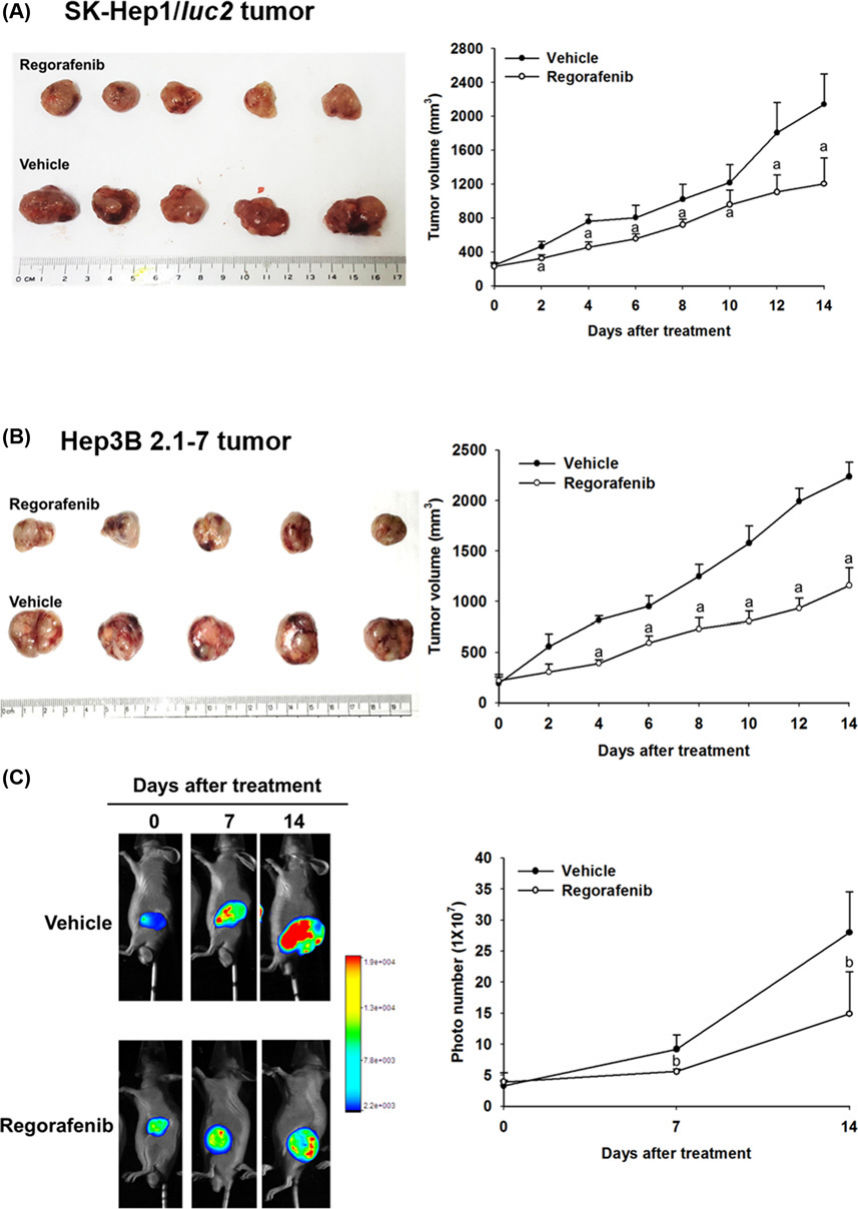
Effect of regorafenib on tumor growth in SK-Hep1/*luc2* and Hep3B 2.1-7 tumor bearing mice Mice were treated with vehicle (140 μl of PBS plus 10 μl of DMSO per day) or regorafenib (20 mg/kg/day) by gavage for 14 days. Tumor growth was evaluated with digital caliper and BLI. (**A**) SK-Hep1/*luc2* tumor volume was measured by digital caliper on day 0, 2, 4, 6, 8, 10, 12, and 14. (**B**) Hep3B 2.1-7 tumor volume was measured by digital caliper on day 0, 2, 4, 6, 8, 10, 12, and 14. (**C**) SK-Hep1/*luc2* tumor growth was monitored with BLI on day 0, 7, and 14; ^a^*P*<0.01 and ^b^*P*<0.05 as compared with vehicle group. BLI, bioluminescence imaging; DMSO, dimethyl sulfoxide; PBS, phosphate-buffered solution.

Mice bearing two different HCC cell lines SK-Hep1/*luc2* and Hep3B 2.1-7 were prepared for animal studies respectively. The study procedure of animal was displayed as Figure 1. We used the digital caliper and BLI to evaluate the effect of regorafenib on tumor growth in SK-Hep1/*luc2* and Hep3B 2.1-7 tumor bearing mice in the experiment. We found regorafenib significantly inhibited both SK-Hep1/*luc2* and Hep3B 2.1-7 tumor growth as compared with vehicle group (Figure 2A,B). The quantification result of tumor volume also showed marked suppression by regorafenib treatment in a SK-Hep1/*luc2* and Hep3B 2.1-7 bearing model. In SK-Hep1/*luc2* group, we also confirmed that photons emitted from the tumors of vehicle group were significantly higher than that of regorafenib group in Figure 2C. These results suggested that regorafenib effectively inhibited HCC tumor growth.

### Regorafenib inhibits expression of ERK/NF-κB-modulated downstream effector proteins and triggers expression of apoptotic proteins in SK-Hep1/luc2 and Hep3B 2.1-7 tumor bearing mice

**Figure 3:**
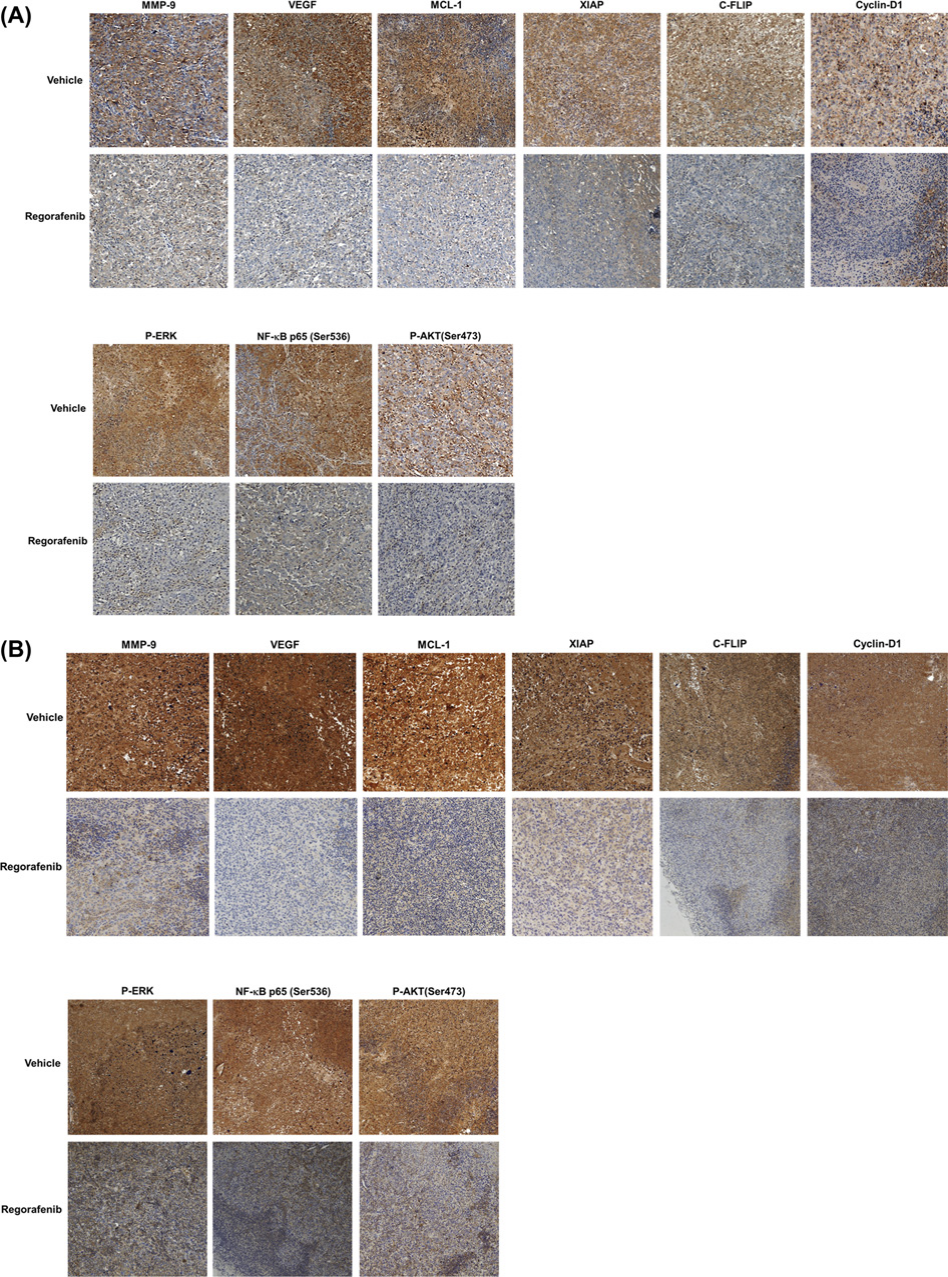

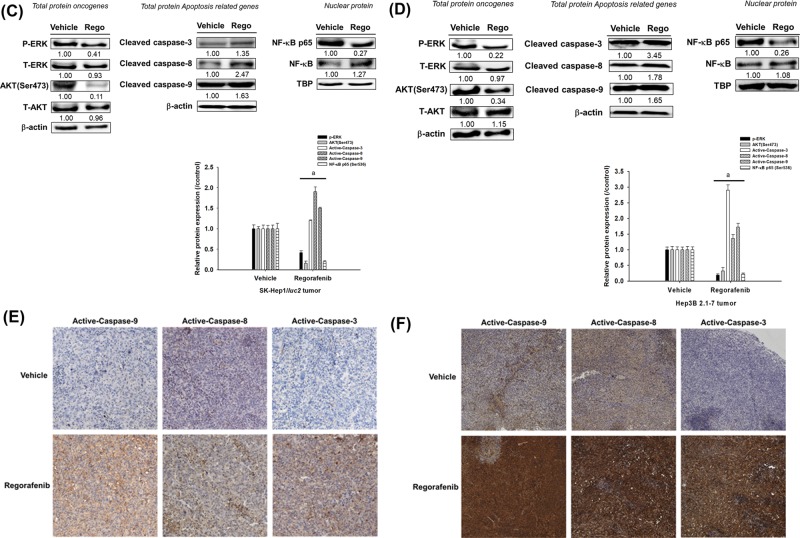
Effect of regorafenib on expression of P-ERK, AKT (Ser473), NF-κB p65 (Ser473), and NF-κB-modulated downstream effector proteins in SK-Hep1/*luc2* tumor and Hep3B 2.1-7 bearing mice Mice were killed on day 14 after treatments and protein levels in tumor tissues were evaluated with IHC staining. (**A**) Protein levels of MMP-9, VEGF, MCL-1, XIAP, C-FLIP, Cyclin-D1, and P-ERK, AKT (Ser473), NF-κB p65 (Ser473) on SK-Hep1/*luc2* tumor by IHC. (**B**) IHC staining of Hep3B 2.1-7 tumor. (**C**) Phosphorylation oncogenes and apoptosis-related cleavage proteins expression which validated by Western blotting on SK-Hep1/*luc2* tumor. (**D**) Western blotting of Hep3B 2.1-7 tumor. (**E**) Expression of antiapoptotic proteins (active Capase-9, -8, and -3) on SK-Hep1/*luc2* tumor by IHC. (**F**) IHC staining of Hep3B 2.1-7 tumor; ^a^*P*<0.01 as compared with vehicle group; C-FLIP, cellular FADD-like IL-1β-converting enzyme-inhibitory protein; IHC, immunohistochemistry; MCL, myeloid leukemia cell differentiation protein; MMP, matrix metallopeptidase; NF-κB, nuclear factor-κB; P-ERK, phosphorylated extracellular signal-regulated kinase; VEGF, vascular endothelial growth factor; XIAP, X-linked inhibitor of apoptosis protein.

In order to measure the mechanism of regorafenib, we measured different types of proteins expression by IHC and *ex vivo* Western blotting. In Figure 3A,B IHC results, regorafenib significantly suppressed protein expression of MMP-9, VEGF, MCL-1, XIAP, C-FLIP, Cyclin-D1, P-ERK (Thr202/Tyr204), NF-κB p65 (Ser536), and AKT (Ser473), as compared with vehicle group. The inhibition of phospho-ERK, phospho-AKT, and NF-κB p65 proteins expression was double confirmed with *ex vivo* Western blotting assay (Figure 3C,D). After regorafenib treatment, the expression level of cleaved caspase-3, cleaved caspase-8, and caspase-9 was increased in two types of HCC model (Figure 3C,D). In addition, our IHC results also suggested that regorafenib may significantly induce expression of active Caspase-3, -8, and -9 as compared with vehicle group (Figure 3E,F). In sum, our results represented that regorafenib enhanced treatment efficacy of HCC via suppression of ERK/NF-κB signaling pathway.

### General toxicity analysis of regorafenib in SK-Hep1/luc2 and Hep3B 2.1-7 tumor bearing mice

**Figure 4:**
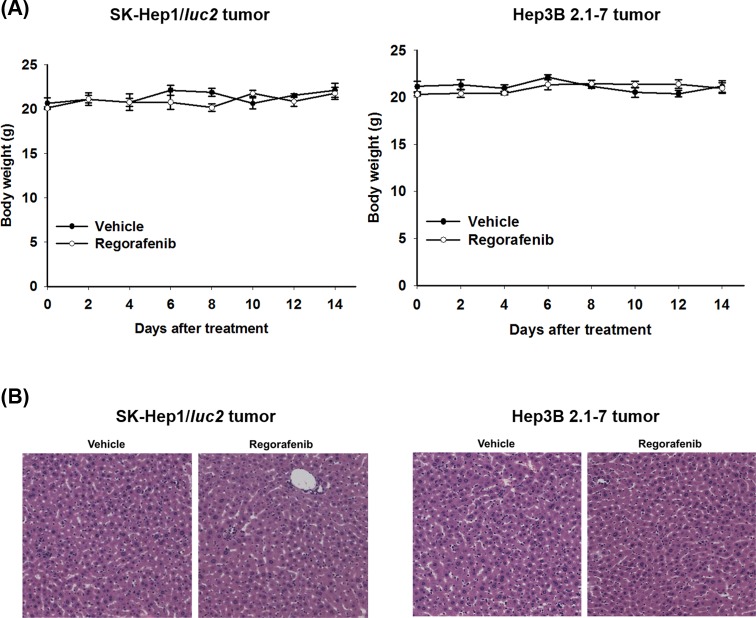
Toxicity investigation of regorafenib in SK-Hep1/*luc2* and Hep3B 2.1-7 tumor bearing mice Toxicity of regorafenib was evaluated with body weight measurement and pathological examination of mice livers. (**A**) Body weight of both HCC bearing mouse were measured on day 0, 2, 4, 6, 8, 10, 12, and 14. (**B**) Mice were killed on day 14 after treatments and pathological examination of livers was evaluated with H&E staining; HE, hematoxylin and eosin.

Body weight measurement and pathological examination of liver were used to monitor general toxicity of regorafenib in SK-Hep1/*luc2* and Hep3B 2.1-7 tumor bearing mice. Figure 4A presented no significant difference in body weight between regorafenib and vehicle groups. We also could not identify any obvious difference in liver morphology between two groups in SK-Hep1/*luc2* and Hep3B 2.1-7 tumor bearing mice (Figure 4B). Here, we proved that non-general toxicity was occurred after regorafenib administration.

## Discussion

High expression of NF-κB activation, which plays key mediator of tumor progression, is associated with poor survival in HCC patients [14]. NF-κB target gene encoding proteins such as MMP-9, VEGF, MCL-1, XIAP, C-FLIP, and Cyclin-D1, promote metastasis, angiogenesis, antiapoptosis, and proliferation in HCC leading to tumor progression [4,7]. High protein levels of MMP-9, VEGF, MCL-1, XIAP, C-FLIP, and Cyclin-D1 have been suggested as biomarkers for poor prognosis and these proteins are potential therapeutic targets for the development of anticancer treatments in patients with HCC [15–18]. In the previous study, we found regorafenib, as inhibitor of NF-κB signaling, suppressed tumor growth and metastatic potential in HCC *in vitro* [4]. However, whether regorafenib down-regulates NF-κB-modulated tumor progression in HCC *in vivo* is ambiguous. Therefore, we used SK-Hep1/*luc2* and Hep3B 2.1-7 tumor bearing mice to verify the effect of regorafenib on NF-κB-modulated tumor progression in HCC *in vivo*.

Regorafenib, a multikinase inhibitor, is used to treat different kinds of cancer. Su et al. [19] found regorafenib reduced protein expression of VEGF through suppression of signal transducer and activator of transcription 3 (STAT-3) activation in triple negative breast cancer cells *in vitro* and *in vivo*. Chen et al. [20] found regorafenib triggered p53-upregulated modulator of apoptosis (PUMA)-mediated apoptosis through induction of NF-κB pathway in colorectal cancer *in vitro* and *in vivo*. Tai et al. presented regorafenib induced apoptosis and inhibited expression levels of antiapoptotic and proliferative proteins (MCL-1, Survivin, and Cyclin-D1) via inhibition of STAT-3 activation. Tai et al. also presented tumor size was significant diminished by regorafenib treatment (20 mg/kg/day) as compared with vehicle group in HCC PLC tumor bearing mice [21]. In the present study, we began with finding the same dose of regorafenib as used by Tai et al. also significantly reduced tumor growth as compared with vehicle group in SK-Hep1/*luc2* and Hep3B 2.1-7 tumor bearing mice (Figure 2A,B). Next, we used *ex vivo* Western and IHC staining to verify the effect of regorafenib on NF-κB-modulated tumor progression in SK-Hep1/*luc2* and Hep3B 2.1-7 tumor bearing mice. The result presented regorafenib significantly reduced protein levels of P-ERK, P-AKT, NF-κB p65 (Ser536), MMP-9, VEGF, MCL-1, XIAP, C-FLIP, and Cyclin-D1 (Figure 3A–C).

Rapidly accelerated fibrosarcoma (RAF)/mitogen-activated protein kinase kinase (MEK)/ERK and phosphoinositide 3-kinase (PI3K)/AKT signaling cascades both play critical roles in the progression of HCC. NF-κB activation can be regulated by P-ERK and AKT in cancer cells [7,22]. In previous study, we presented regorafenib induced extrinsic and intrinsic apoptosis through inhibition of ERK/NF-κB activation in SK-Hep1 *in vitro* [7]. In contrast, Chen et al. [20] presented regorafenib inhibited ERK phosphorylation and induced expression of active-NF-κB, Caspase-3, -8, and -9 in colorectal cancer cells. Therefore, we performed the current *in vivo* study and Figure 3A–E indicated regorafenib not only reduced ERK, NF-κB, and AKT phosphorylation but also triggered expression of active Caspase-9, -8, and -3 in SK-Hep1/luc2 and Hep3B 2.1-7 tumor bearing mice. All these findings suggested mechanism of regorafenib anticancer effect in HCC *in vivo* is similar to that observed *in vitro*.

In conclusion, the present study demonstrated regorafenib inhibited tumor progression through suppression of ERK/NF-κB activation in HCC *in vivo*. We performed experiment on two different HCC bearing mice and suggested anticancer effect of regorafenib is mediated by the inhibition of ERK/NF-κB activation in HCC *in vivo*.

## Abbreviations

BLI: bioluminescence imaging
C-FLIP: cellular FADD-like IL-1β-converting enzyme-inhibitory protein
ERK: extracellular signal-regulated kinase
HCC: hepatocellular carcinoma
IHC: immunohistochemistry
MCL-1: induced myeloid leukemia cell differentiation protein
MMP9: matrix metallopeptidase
NF-κB: nuclear factor-κB
TBP: TATA-binding protein
VEGF: vascular endothelial growth factor
